# Penile strangulation by metallic key holder in an adolescent: a case report and literature review

**DOI:** 10.1097/MS9.0000000000004946

**Published:** 2026-04-09

**Authors:** Anas R. Tuqan, Tareq Jarrar, Tariq Asi

**Affiliations:** Faculty of Medicine, Al-Quds University, Abu-Dis, East Jerusalem, Palestine

**Keywords:** adolescent, case report, metallic key holder, penile strangulation, surgical removal

## Abstract

**Introduction and importance:**

Penile strangulation is an uncommon urological emergency in which an external constricting object initially impedes venous and lymphatic outflow and, if prolonged, leads to arterial insufficiency and tissue necrosis.

**Case presentation:**

A 16-year-old male presented after approximately 2 weeks of progressive penile swelling and pain following self-application of a metallic key holder at the midshaft. Examination revealed marked distal edema, coronal congestion, shaft erythema, focal ulceration, and areas of skin necrosis, with preserved distal sensation and no urinary retention. Clinical assessment suggested adequate distal perfusion without deep structural compromise. Under general anesthesia, the constrictor was divided and removed, necrotic tissue was debrided, a drain was placed, and prophylactic antibiotics were administered. At the 2-week follow-up, the wound had healed without infection; voiding and erectile function were preserved, and a psychiatric referral was arranged.

**Clinical discussion:**

Injury severity is shaped by the duration of constriction, the device used, and the degree of preserved perfusion. Stepwise management from bedside techniques to operative or high-power cutting tools guides safe removal. Early decompression typically maintains urinary and sexual function, whereas delayed intervention increases the risk of stricture, erectile dysfunction, and gangrene. Coordinated surgical management and psychiatric follow-up are essential to optimize outcomes and reduce recurrence.

**Conclusion:**

Penile strangulation demands urgent, device-specific intervention guided by assessment of constriction duration, perfusion status, and object composition. Adherence to graded management algorithms and early psychiatric evaluation are recommended to minimize long-term morbidity and address contributory behavioral factors.

## Background

Penile strangulation is a challenging but rare urologic emergency caused by a constricting object over the penis, producing venous and lymphatic outflow obstruction and eventually arterial compromise[1]. The causes vary with age as well as with intent. In general, both children and adults can be affected by intrinsic psychological or behavioral disorders during such events^[^2–4^]^. The objects used in penile strangulation are wide-ranging, including metallic and nonmetallic objects such as rings, bands, and pipes[3]. The clinical presentation ranges from mild distal edema to extensive ischemia and necrosis. The extent of injury primarily depends on constriction time, object type (metallic or nonmetallic), and the degree of tightness[2]. Numerous removal methods have been reported in the literature, ranging from simple manual removal to cutting tools and dedicated mechanical devices. Nonmetallic foreign bodies tend to be simpler to remove, with metallic devices being more complicated and often requiring surgical removal under anesthesia^[^3,5^]^. Ultimately, early diagnosis and proper management of any underlying psychological derangement are crucial for positive outcomes[4]. Here, we report our management of a case of penile strangulation caused by a metallic key holder in a 16-year-old adolescent. This work has been reported in line with the SCARE 2025 criteria[6].

**HIGHLIGHTS**

- Timely removal of the constricting object typically preserves urinary and sexual function, whereas delayed intervention increases the risk of stricture, erectile dysfunction, and tissue loss.
- Management should follow a stepwise, device-specific algorithm – starting with minimally invasive bedside methods and escalating to operative or high-power cutting tools when needed.
- Psychiatric assessment is an important component of care, as many cases involve behavioral, psychological, or autoerotic factors and may recur without appropriate follow-up.

## Case presentation

A 16-year-old male adolescent presented with progressive penile swelling and pain. He had been referred from the emergency department with an initial working diagnosis of paraphimosis. The patient’s medical history was unremarkable. The patient declined to provide detailed information regarding the circumstances of the injury; a delay in presentation attributable to embarrassment and the possibility of self-inflicted placement of the constricting device in the context of masturbation or sexual curiosity were therefore considered. He reported that the metallic key holder had been applied approximately 2 weeks prior to presentation.

**Figure 1.**
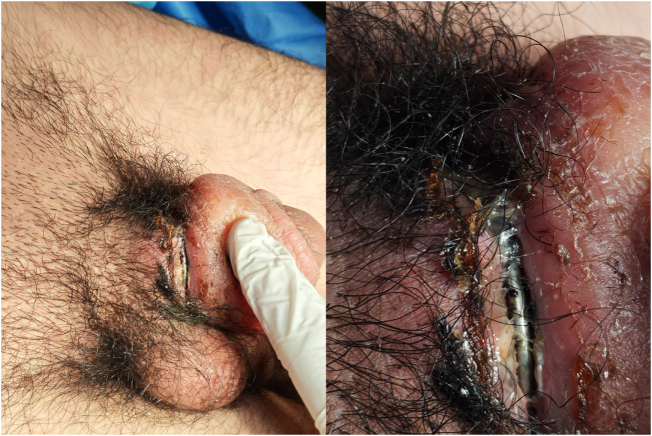
A metallic key holder encircling the midshaft of the penis at presentation.

On examination, the patient was hemodynamically stable. A metallic key holder encircled the midshaft of the penis. The findings included marked distal edema, coronal congestion, erythema, focal shaft ulceration, and areas of necrotic skin (Fig. 1). Distal sensation was preserved, and there was no urinary retention. Clinical assessment suggested adequate distal perfusion without evidence of deep tissue compromise. According to the Bhat classification, the injury was categorized as Grade II.

**Figure 2.**
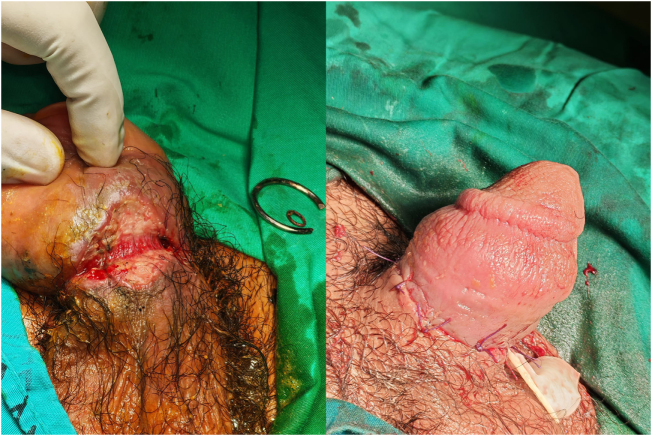
Intraoperative view after removal of the metallic ring, showing an approximation of viable skin edges with interrupted sutures.

The patient was managed as a surgical emergency. Imaging and laboratory investigations were not performed because bedside assessment indicated preserved distal perfusion and no signs of deep structural injury. Manual extraction methods proved impractical because of the rigidity and tightness of the metallic constrictor; therefore, definitive surgical intervention was undertaken. Under general anesthesia, the ring was divided and removed with a wire cutter. Necrotic tissue was debrided, and viable skin edges were approximated with interrupted sutures (Fig. 2). Intraoperative inspection revealed grossly intact corpora cavernosa and urethra, with Buck’s fascia delineating the inner limit of tissue injury. A wound swab for culture was obtained, and a Penrose drain was placed. Prophylactic intravenous cefazolin was administered intraoperatively.

The postoperative course was uncomplicated. The drain was removed on the first postoperative day, and the patient was discharged on oral amoxicillin, diazepam, and analgesics. At the 2-week follow-up, the wound demonstrated satisfactory healing without clinical evidence of infection; the patient reported normal voiding and preserved erectile function. Referral to psychiatric services was arranged for further assessment and support.

## Discussion

Penile strangulation is an uncommon urological emergency that was first described by Gauthier in 1755[7]. The motives for applying constricting foreign bodies to the external genitalia vary by age: in middle-aged and older men, the practice is most often related to attempts to enhance sexual performance or to autoerotic behavior, whereas in adolescents, it is more commonly associated with masturbation and sexual curiosity[8]. Some Asian countries have observed penile constriction as a result of the routine application of elastic bands to control urinary frequency or incontinence[4]. A substantial proportion of affected individuals have coexisting psychiatric disorders, and many delay presentation because of embarrassment or only present after unsuccessful attempts at self-removal[9]. Reported constricting agents encompass a broad spectrum of improvised items, including metallic objects (e.g., iron or steel rings, nuts, hammerheads, wedding rings, and iron pipes) and nonmetallic materials (e.g., bottles of various sizes, rubber bands, and hair tourniquets)[10].

The sequence of tissue injury in penile strangulation is largely determined by the duration of constriction and the degree of residual perfusion. Initially, venous and lymphatic outflow distal to the constricting device is impeded, producing distal edema, congestion, and engorgement. Progressive compression compromises nutritive blood flow to the skin and superficial tissues, whereas deeper erectile structures (corpora cavernosa and corpus spongiosum) may maintain arterial perfusion longer because of their higher intracavernosal pressures. With prolonged constriction, arterial inflow becomes increasingly impaired, resulting in tissue ischemia; the skin over the constricted segment evolves from erythema to ulceration and may progress to full-thickness ischemic necrosis and gangrene. Typically, in long-standing cases (weeks to months), autoamputation of the distal penis has been reported^[^11,12^]^. In the present patient, the preservation of distal sensation after 2 weeks of constriction suggested that deep neural elements and the arterial supply to the principal erectile tissues remained functionally intact, despite marked ischemic injury of the superficial tissues manifesting as necrosis and ulceration.

Bhat and colleagues proposed a grading system for penile incarceration that correlates clinical findings with severity: Grade 1 – distal edema only; Grade 2 – distal edema with skin and urethral trauma, compression of the corpus spongiosum, and decreased distal sensation; Grade 3 – skin and urethral trauma with absent distal sensation; Grade 4 – separation of the corpus spongiosum with urethral fistula, compression of the corpora cavernosa, and absent distal sensation; and Grade 5 – gangrene, necrosis, or distal penile amputation[13]. The combination of epidermal ulceration/necrosis with apparent preservation of deeper structures in this case is most consistent with Grade 2 injury, which aligns with reports that more severe sequelae are less likely when deep corporal structures remain perfused.

The management algorithms described by Puvvada *et al* employ a structured, three-tiered approach aimed at rapid, safe decompression while maximizing tissue preservation. Level 1 comprises bedside, minimally invasive measures under penile block anesthesia – generous lubrication and manual removal, the string technique with or without corporal blood aspiration via an 18-G needle, and the cutting of thin nonmetallic constrictors with heavy surgical scissors. Failure of these measures necessitates escalation to Level 2, which involves controlled operative intervention under spinal or general anesthesia via orthopedic and surgical instruments (e.g., bone cutters, K-wire cutters, oscillating saws, and dental micromotors) to divide thicker nonmetallic or slender metallic devices while minimizing iatrogenic injury. Level 3 addresses resistant or heavy metallic constrictors and requires high-power cutting tools (orthopedic drills, pedal cutters, angle grinders, reciprocating saws) together with meticulous protective measures – adequate draping, the placement of malleable retractors beneath the object, stabilization with Allis forceps, and continuous cooling irrigation – to avoid thermal and mechanical damage[12].

When decompression is achieved promptly and appropriately, the prognosis is generally favorable. Long-term complications such as urethral stricture or erectile dysfunction are uncommon but can occur, particularly after prolonged ischemia. Psychiatric assessment and follow-up are frequently indicated in cases associated with autoerotic behavior or underlying mental health disorders to address contributory factors and reduce the risk of recurrence[14].

## Conclusion

Timely, device-specific decompression guided by clinical assessment of perfusion can preserve urinary and sexual function even after prolonged constriction. The implementation of graded management algorithms, clinician training in device-appropriate techniques, and routine psychiatric assessment are recommended to optimize outcomes and reduce recurrence. Limitations include the single-case design; systematic studies are needed to refine prognostic indicators and long-term sequelae.

## Data Availability

The data that support the findings of this study are available from the corresponding author upon reasonable request.

## Assistance with the study

The authors declare that no external assistance was received in the conduct of this study.

## Presentation

The authors confirm that this work has not been previously presented in any form.
